# The Significance of Right‐Sided Precordial ECG Leads (V3R and V4R) in Assessing Right Ventricular Dysfunction: A Single Center Cross‐Sectional Study

**DOI:** 10.1111/anec.70006

**Published:** 2024-09-09

**Authors:** Reza Khosravi, Hasan Shemirani, Marziyeh Najafi, Zahra Ghaffarinejad, Mahta Arbabi, Marzieh Tajmirriahi

**Affiliations:** ^1^ Isfahan University of Medical Sciences Isfahan Iran; ^2^ Cardiac Electrophysiology Research Center, Rajaie Cardiovascular Medical and Research Center Iran University of Medical Sciences Tehran Iran; ^3^ Echocardiography Research Center, Rajaie Cardiovascular Medical and Research Center Iran University of Medical Sciences Tehran Iran

**Keywords:** ECG, precordial leads, right ventricle, systolic dysfunction, V3R, V4R

## Abstract

**Background:**

Right ventricular systolic dysfunction is associated with poor prognosis and increased mortality rates. Our objective was to investigate ECG changes in patients with this condition, focusing on the right‐sided precordial leads.

**Methods:**

In this cross‐sectional study, 60 patients with right ventricular dysfunction were included from April 2020 to April 2021. Cardiac structure and function were assessed using 2D transthoracic echocardiography. Standard 12‐lead electrocardiograms and right‐sided precordial ECGs (V3R‐V4R) were obtained and analyzed for QRS complex configuration, ST‐segment elevation, and T‐wave morphology.

**Results:**

In our study, the majority were male (70.0%) with a mean age of 58.76 years. The most common initial diagnoses were pulmonary thromboembolism (43.3%), chronic obstructive pulmonary disease (26.7%), and pulmonary hypertension (25.0%). The predominant ECG finding in the right‐sided precordial leads (V3R, V4R) was a deep negative T wave (90.0%). Patients with severe right ventricular systolic dysfunction often exhibited a qR pattern (41.2%), whereas those with nonsevere dysfunction showed rS and QS patterns (55.8%). Approximately 41.0% of severe RV dysfunction cases had ST segment depression in the right‐sided precordial leads, and 28.0% of patients displayed signs of right atrial abnormality.

**Conclusion:**

The study found that qR, rS, and QS patterns were more prevalent in V3R and V4R leads among patients with severe and nonsevere right ventricular systolic dysfunction. The most common ECG feature observed was deep T‐wave inversion in these leads. The study recommends using right‐sided precordial leads in all patients with RV systolic dysfunction for early detection and risk stratification.

## Graphical abstract

This study assesses the utilization of V3R and V4R right‐sided precordial ECG leads in cases of right‐sided ventricular dysfunction.

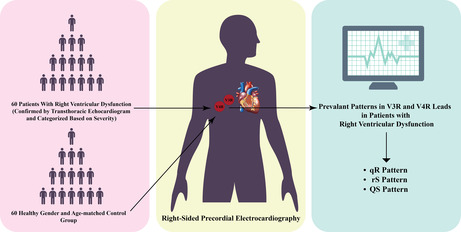

Right ventricular failure occurs when the right ventricle (RV) is unable to maintain sufficient cardiac output despite adequate preload (1). This dysfunction can be caused by increased RV afterload, decreased RV contractility, or a combination of both factors. Critically ill patients often experience RV failure, particularly because of conditions such as pulmonary embolism, pulmonary arterial hypertension (PAH), acute respiratory distress syndrome (ARDS), and sepsis (2). The presence of RV dysfunction is strongly associated with a poor prognosis and high rates of morbidity and mortality (Golpe et al. 2010).

In clinical practice, 2D transthoracic echocardiography (2D TTE) is commonly used to noninvasively evaluate RV dysfunction. However, ECG can also be a valuable tool for assessing a patient's prognosis and guiding further diagnostic and therapeutic decisions. ECG offers advantages such as simplicity, widespread availability, and low cost, making it particularly useful in emergency triage situations (Vanni et al. 2009). While right‐sided precordial ECG leads (V3R, V4R) have traditionally been used to detect right ventricular myocardial infarction (RVMI), there is currently no research on the ECG changes in these leads specifically in patients with RV dysfunction.

Therefore, the objective of this study is to investigate the ECG changes in the right‐sided precordial leads (V3R, V4R) among patients with RV dysfunction. To the best of our knowledge, this is the first study to assess these ECG changes in this specific patient population.

## 1 Methods

### 1.1 Setting and Design

This cross‐sectional study was conducted at the Khorshid Hospital, which is affiliated with the Isfahan University of Medical Sciences (IUMS). This study aimed to evaluate patients with right ventricular (RV) dysfunction over 1 year. The evaluation period for this study was from April 3, 2020 to April 4, 2021. All eligible patients who were admitted to the hospital during this period with a diagnosis of RV systolic dysfunction were included in the study (convenience sampling). RV systolic dysfunction was confirmed by using 2D TTE based on the recommendations of the American Society of Echocardiography for cardiac chamber quantification in adults (Lang et al. 2015). Echocardiography was performed using standard views, with the patient in the left lateral decubitus position, using a Philips EPIQ 7 cardiovascular ultrasound machine. A single experienced examiner, who had completed a fellowship in echocardiography, obtained conventional echocardiographic images and cine loops of all patients using an M5S transducer.

Furthermore, a total of 60 sex‐ and age‐matched controls with normal echocardiography were included for better comparison.

### 1.2 Inclusion and Exclusion Criteria

The inclusion criteria for this study were as follows: patients aged 18 years or older with a confirmed diagnosis of RV systolic dysfunction, and patients who provided formal consent to participate and complete the study. RV systolic dysfunction was defined using 2D TTE based on the presence of one or more of the following criteria: tricuspid annular plane systolic excursion (TAPSE) <1.6 cm, basal RV free wall velocity (*S*′) <10 cm/s, or RV fractional area change (FAC) <0.35 (Jaff 2011; Lang et al. 2015; Rudski et al. 2010). Patients with a body mass index (BMI) of 18 or less, skeletal anomalies, such as pectus excavatum, and those with incomplete information were excluded from the study. A total of 60 eligible patients were selected to participate.

### 1.3 Data Collection

Using a checklist, a general physician obtained the data. All completed questionnaires were thoroughly checked and verified for errors before the final analysis. Measurements of RV systolic function were taken, including TAPSE, RV fractional area change (FAC), and RV free wall velocity (*S*′). Left ventricular ejection fraction (LVEF), diastolic function, pulmonary artery pressure (PAP), pulmonary acceleration time, and right atrial (RA) size were also measured according to guidelines (Lang et al. 2015).

During the study, a standard 12‐lead ECG and two right‐sided precordial leads (V3R‐V4R) were recorded from participants in a supine position at rest, with a paper speed of 25 mm/s and calibration of 1 mV/10 mm. The standard 12‐lead ECG findings, including axis, rhythm, rate, P wave, PR interval, QRS width, and R‐wave progression in precordial leads, were analyzed. Additionally, the findings from the two right‐sided precordial leads (V3R, V4R) were examined, including P wave, QRS width, QRS pattern, QRS voltage, ST‐segment, and T wave. If a patient exhibited serial ECG changes, all ECG patterns were analyzed.

### 1.4 Statistical Analysis

Data were analyzed using descriptive statistics including mean ± standard deviation (SD), median, frequencies, and percentages wherever applicable. Differences between subgroups were assessed using a one‐way analysis of variance (ANOVA) for continuous and normally distributed variables and a chi‐square test (or Fisher's exact test) for categorical variables. A test was considered statistically significant if the probability value (*p*‐value) was <0.05. All analyses were carried out using Stata software (version 14.1; Stata Corp, College Station, TX, USA).

### 1.5 Ethics Statement

The study protocol (Ethics No. IR.MUI.MED.REC.1399.626) was approved by the Research Ethics Committee at Isfahan University of Medical Sciences (IUMS). Prior to participating in the study, patients were informed about the study and provided their consent by signing a consent form. The confidentiality of patient data was strictly maintained, with access limited to the researchers involved in the study.

## 2 Results

Table 1 presents the basic characteristics of the patients. Out of 60 patients, 18 (30.0%) were female and 42 (70.0%) were male, with an average age of 58.76 ± 2.29 years (ranging from 20 to 87). Fifteen (25.0%) patients had diabetes, 23 (38.3%) had hypertension, 10 (16.7%) were active smokers, and 14 (23.3%) had hyperlipidemia. The most common initial diagnoses were pulmonary thromboembolism (PTE), chronic obstructive pulmonary disease (COPD), and pulmonary hypertension, accounting for 43.3%, 26.7%, and 25.0% of cases, respectively.

**TABLE 1:** The baseline characteristics of patients (*n* = 60).

Characteristics	
Age (years)	58.76 ± 2.29
Gender (male, %)	42 (70.0)
Diabetes mellitus	15 (25.0)
Hypertension	23 (38.3)
Hyperlipidemia	14 (23.3)
Current smoker	10 (16.7)
BMI (kg/m^2^)	26.45 ± 4.18
First diagnosis	
COPD	16 (26.7)
PTE	26 (43.3)
Bronchiectasis	1 (1.7)
Pulmonary hypertension	15 (25.0)
RV failure	2 (3.3)

Abbreviations: BMI, body mass index; COPD, chronic obstructive pulmonary disease; *N*, number; PTE, pulmonary thromboembolism.

Out of 60 patients, 53 (88.3%) had mild and moderate TR, and 43 (71.7%) had mild MR. The average LVEF was 47.53 ± 0.59%. The echocardiographic characteristics are detailed in Table 2.

**TABLE 2:** The echocardiography characteristics of patients (*n* = 60).

Characteristics	*N* (%)
TAPSE (mm)	31.0 ± 12.53
FAC (%)	30.0 ± 22.48
PAP (mmHg)	52.2 ± 62.56
RA area (cm^2^)	0.62 ± 23.2
Pulmonary acceleration time (ms)	1.90 ± 75.1
LVEF (%)	47.53 ± 0.59
RV free wall velocity (*S*′) (cm/s)	0.18 ± 8.47
LV diastolic dysfunction grade	
1	57 (95.0)
2	3 (5.0)
Tricuspid regurgitation	
Mild	27 (45.0)
Moderate	26 (43.3)
Severe	7 (11.7)
Mitral regurgitation	
Mild	43 (71.7)
Moderate	14 (23.3)
Severe	3 (5.0)

Abbreviations: FAC, fractional area change; LV, left ventricle; LVEF, left ventricular ejection fraction; *N*, number; PAP, pulmonary artery pressure; RA, right atrial; TAPSE, tricuspid annular plane systolic excursion.

**FIGURE 1:**
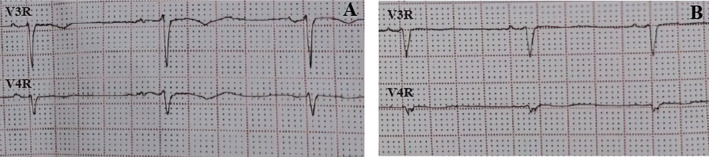
ECG of the healthy control group. rS pattern and inverted T wave are seen in (A), and qS and positive T wave can be seen in (B).

Out of the 60 patients, 21 (35.0%) exhibited a right axis deviation, while only two (3.3%) showed a left axis deviation. The majority, 52 (86.7%), were in normal sinus rhythm, and 8 (13.3%) presented with atrial fibrillation. Additionally, 17 (28.3%) patients had a right atrial abnormality, and 3 (5.0%) had a left atrial abnormality. Narrow QRS widths were observed in 52 (86.7%) cases, and wide QRS widths (RBBB pattern) were seen in 8 (13.3%). The comparison of ECG changes between patients with RV systolic dysfunction and the healthy control group showed significant differences in axis, rhythm, P wave, QRS pattern in right‐sided precordial leads, R wave progression, ST segment, and T wave (*p*‐value < 0.05). Two samples of ECGs of the healthy control group can be seen in Figure 1. Patients with severe RV systolic dysfunction were more likely to exhibit a qR pattern in right‐sided precordial leads (V3R, V4R; 41.2%), while those with nonsevere RV systolic dysfunction were more likely to show rS and QS patterns in V3R, V4R (55.8%; *p* = 0.0001). ST depression was significantly more common in patients with severe RV systolic dysfunction than in those with nonsevere RV systolic dysfunction in right‐sided precordial leads (V3R, V4R; 41.0% vs. 20.9%, *p* = 0.0001). Among the 60 patients with RV systolic dysfunction, deep T wave inversion was the most frequent ECG sign in right‐sided precordial leads (V3R, V4R; 90%). These findings are summarized in Table 3.

**TABLE 3:** Assessing the standard 12 ECG leads and two right‐sided precordial ECG leads based on the severity of RV dysfunction.

Characteristic	Without RV dysfunction		With RV dysfunction		*p*
	Control (*N* = 60)	*p*	Nonsevere (*N* = 43)	Severe (*N* = 17)	
Axis					
Normal	53 (88.3)	<0.001	19 (44.2)	6 (35.3)	0.814
RAD	1 (1.7)		15 (34.9)	6 (35.3)	
LAD	3 (5)		1 (2.3)	1 (5.9)	
Undetermined	3 (5)		8 (18.6)	4 (23.5)	
Rhythm					
Atrial fibrillation	2 (3.3)	<0.001	6 (14.0)	2 (11.8)	0.495
Normal sinus rhythm	58 (96.7)		37 (86.0)	15 (88.2)	
P wave					
Normal	56 (93.3)	<0.001	30 (69.8)	10 (58.8)	0.425
LA abnormality	2 (3.3)		1 (2.3)	2 (11.8)	
RA abnormality	2 (3.3)		12 (27.9)	5 (29.4)	
QRS width					
Narrow	56 (93.3)	0.224	37 (86.0)	15 (88.2)	0.856
Wide	4 (6.7)		6 (14.0)	2 (11.8)	
QRS pattern in right‐sided precordial leads					
QS	2 (3.3)	<0.001	12 (27.9)	1 (5.8)	0.001
R	7 (11.7)		6 (14.0)	2 (11.8)	
Rs	5 (8.3)		1 (2.3)	2 (11.8)	
qR	4 (6.7)		11 (25.6)	7 (41.2)	
rS	31 (51.7)		12 (27.9)	5 (29.4)	
rsR′	1 (1.7)		1 (2.3)	0 (0)	
QRS voltage in right‐sided precordial leads			7.13 ± 0.45	7.10 ± 0.31	0.928
R‐wave progression					
Normal	49 (81.7)	0.005	25 (58.2)	9 (52.9)	0.115
Late	3 (5)		9 (20.9)	5 (29.5)	
Early	8 (13.3)		9 (20.9)	3 (17.6)	
ST segment in right‐sided precordial leads					
Normal	58 (96.7)	<0.001	34 (79.1)	10 (59.0)	0.001
Depression	2 (3.3)		9 (20.9)	7 (41.0)	
T wave in right‐sided precordial leads					
Normal	22 (36.7)	<0.001	5 (11.6)	1 (5.9)	0.047
Negative	38 (63.3)		38 (88.4)	16 (94.1)	

Abbreviations: LAD, left axis deviation; *N*, number; *p*, *p* value; RAD, right axis deviation.

Table 4 presents the right‐sided precordial ECG leads, categorized by the initial diagnosis. A significant difference was observed in the frequency of right axis deviation among patients with COPD (62.4%), PTE (30.8%), and PH (6.6%; *p* = 0.033). The occurrence of right atrial abnormality was 43.8% in COPD patients, 11.5% in PTE patients, and 40.0% in PH patients, respectively (*p* = 0.001). ST depression in the right‐sided precordial leads was notably more prevalent in COPD patients (43.8%) compared to those with PTE (19.2%) or PH (20.0%; *p* = 0.001).

**TABLE 4:** Assessing the standard 12 ECG leads, and 2 right‐sided precordial ECG leads based on the first diagnosis.

Characteristics	RV dysfunction						
	Total (*N* = 60)	COPD (*N* = 16)	PTE (*N* = 26)	Bronchiectasis (*N* = 1)	PH (*N* = 15)	RV failure (*N* = 2)	*p*
Axis							
Normal	25 (41.7)	3 (18.8)	14 (53.8)	0 (0)	7 (46.7)	1 (50.0)	0.033
RAD	21 (35.0)	10 (62.4)	8 (30.8)	1 (100.0)	1 (6.6)	1 (50.0)	
LAD	2 (3.3)	0 (0)	2 (7.7)	0 (0)	0 (0)	0 (0)	
Undetermined	12 (20.0)	3 (18.8)	2 (7.7)	0 (0)	7 (46.7)	0 (0)	
Rhythm							
Atrial fibrillation	8 (13.3)	3 (18.8)	2 (7.7)	0 (0)	3 (20.0)	0 (0)	0.562
Normal sinus Rhythm	52 (86.7)	13 (81.2)	24 (92.3)	1 (100)	12 (80.0)	2 (100)	
P wave							
Normal	40 (66.7)	7 (43.8)	23 (88.5)	0 (0)	9 (60.0)	1 (50.0)	0.001
LA abnormality	3 (5.0)	2 (12.4)	0 (0)	1 (100)	0 (0)	0 (0)	
RA abnormality	17 (28.3)	7 (43.8)	3 (11.5)	0 (0)	6 (40.0)	1 (50.0)	
QRS width							
Narrow	52 (86.7)	13 (81.2)	23 (88.5)	1 (100)	13 (86.7)	2 (100)	0.469
Wide	8 (13.3)	3 (18.8)	3 (11.5)	0 (0)	2 (13.3)	0 (0)	
QRS pattern in right‐sided precordial leads							
QS	13 (21.7)	2 (12.4)	5 (19.2)	0 (0)	5 (33.4)	1 (50.0)	0.366
R	8 (13.3)	3 (18.8)	2 (7.7)	1 (100)	1 (6.6)	1 (50.0)	
Rs	3 (5.0)	2 (12.4)	0 (0)	0 (0)	1 (6.6)	0 (0)	
qR	18 (30.0)	7 (43.8)	8 (30.8)	0 (0)	3 (20.0)	0 (0)	
rS	17 (28.3)	2 (12.4)	10 (38.5)	0 (0)	5 (33.4)	0 (0)	
rsR′	1 (1.7)	0 (0)	1 (3.8)	0 (0)	0 (0)	0 (0)	
QRS voltage in right‐sided precordial leads	7.11 ± 0.37	7.06 ± 0.25	7.08 ± 0.40	—	6.99 ± 0.51	7.03 ± 0.25	0.149
R‐wave progression							
Normal	34 (56.7)	5 (31.2)	20 (77.0)	0 (0)	8 (53.3)	1 (50.0)	0.123
Late	14 (23.3)	5 (31.2)	5 (19.2)	0 (0)	4 (26.7)	0 (0)	
Early	12 (20.0)	6 (37.6)	1 (3.8)	1 (100)	3 (20.0)	1 (50.0)	
ST segment							
Normal	44 (73.3)	9 (56.2)	21 (80.8)	1 (100)	12 (80.0)	1 (50.0)	0.001
Depression	16 (26.7)	7 (43.8)	5 (19.2)	0 (0)	3 (20.0)	1 (50.0)	
T wave in right‐sided precordial leads							
Normal	6 (10.0)	2 (12.4)	1 (3.8)	0 (0)	3 (20.0)	0 (0)	0.524
Inversion	54 (90.0)	14 (87.6)	25 (96.2)	1 (100)	12 (80.0)	2 (100)	

Abbreviations: COPD, chronic obstructive pulmonary disease; LAD, left axis deviation; *N*, number; PH, pulmonary hypertension; PTE, pulmonary thromboembolism; RAD, right axis deviation.

## 3 Discussion

**FIGURE 2:**
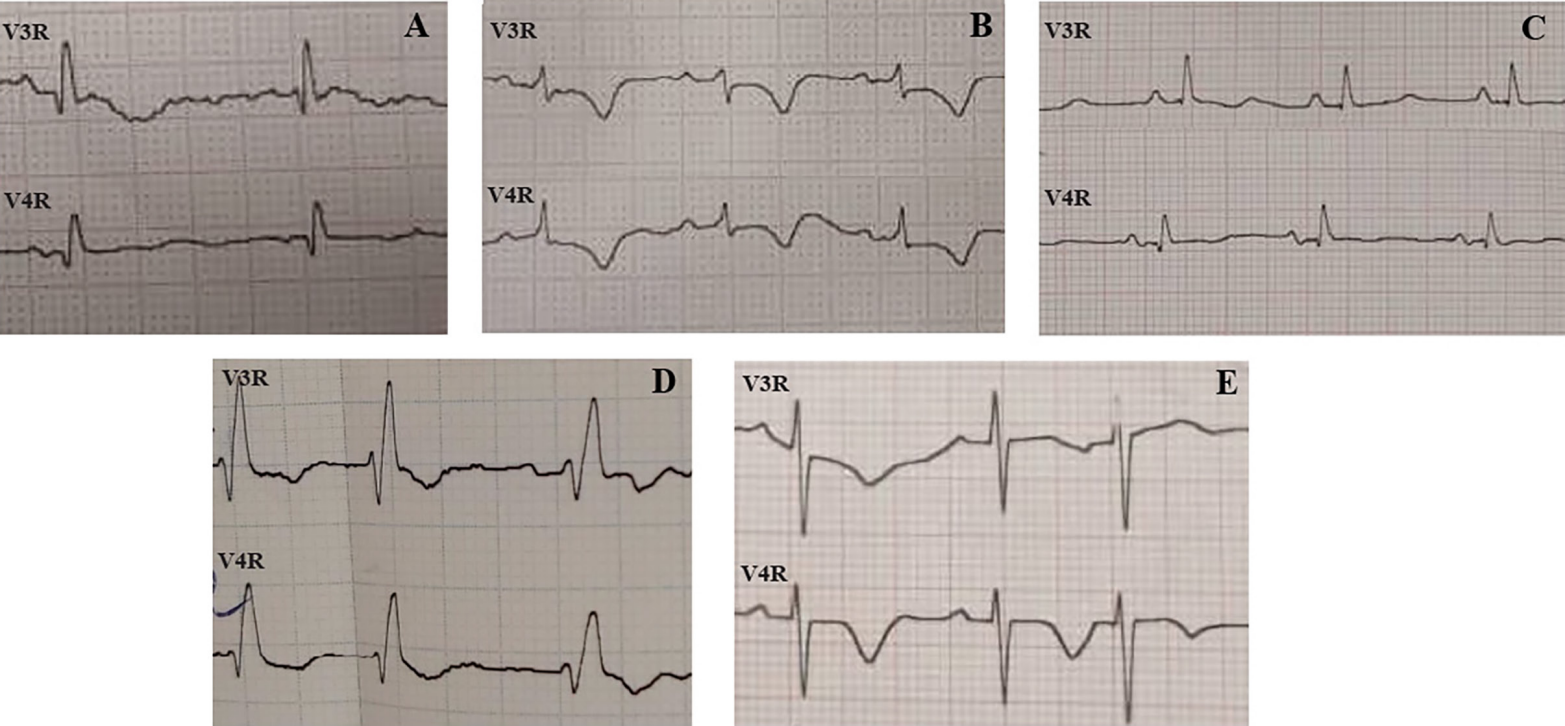
(A, C, and D) Show the qR pattern; and (B, E) demonstrate the rS pattern in V3R and V4R. Alternance of deep T wave inversion can be seen in B, D, and E. RBBB pattern is also notable in D.

This study showed qR pattern in V3R, V4R was significantly most common in patients with severe RV systolic dysfunction in comparison with nonsevere RV systolic dysfunction in which rS and QS patterns in V3R, V4R (Figure 2) were significantly most common in patients with nonsevere systolic RV dysfunction (*p* = 0.0001). The most common ECG feature was deep negative T wave in right precordial leads (V3R, V4R; 90.0%) in both groups. Choi and Park (2012), Vanni et al. (2009), Kim et al. (2009), and Ferrari et al. (1997) demonstrated that the precordial negative T wave was the most frequent ECG feature in acute pulmonary embolism patients with RV systolic dysfunction. Moreover, Zhan and colleagues reported that the most common ECG feature was T wave changes in RV systolic dysfunction patients (Zhong‐qun et al. 2014). Kostrubiec et al. (2010) illustrated that all patients with RV systolic dysfunction showed inverted T‐wave in the precordial leads. Negative T waves were closely associated with changes in RV systolic dysfunction. In other words, improvement in RV systolic dysfunction might be predicted by the disappearance of the negative T wave on precordial leads (Choi and Park 2012).

The mechanism responsible for the appearance of the negative T waves in precordial leads in patients with RV dysfunction may be associated with the development of rapid right ventricular pressure overload and enlargement (Ferrari et al. 1997; Kosuge et al. 2006; Yoshinaga et al. 1999). Some studies have attributed the mechanism of inverted T wave to myocardial ischemia, and the release of various chemical mediators such as catecholamines and histamine (Geibel et al. 2005; Meyer, Planquette, and Sanchez 2008; Sarin, Elmi, and Nassef 2005).

Early detection of high‐risk patients with RV dysfunction by identifying inverted T waves in precordial leads on admission has significant therapeutic implications. Additionally, predicting the improvement of RV dysfunction by the disappearance of the inverted T wave is important, because persistent RV dysfunction is related to recurrent thromboembolic occurrences (Choi and Park 2012; Kim et al. 2009). Grifoni et al. (2006) found that persistent RV systolic dysfunction at hospital discharge occurred in nearly 20% of patients. Therefore, patients with persistent RV dysfunction at discharge should receive strict surveillance for recurrence.

Right axis deviation occurred in 35% of cases. The preexisting disease may also impact the axis deviation on presentation ECG. As we reported PTE was the most frequent diagnosis in patients with RV dysfunction (43.3%). Right axis deviation was described as the classic axis change associated with PTE (Nielsen et al. 1989; Panos et al. 1988; Sreeram et al. 1994).

This study showed that 28.3% of patients had right atrial abnormality and 5.0% of patients had left atrial abnormality. An increase in the P wave amplitude >2.5 mV, known as right atrial abnormality P wave, has been typically associated with RV dysfunction and PTE, consequently resulting from RV hypertrophy or enlargement and/or associated with acute obstruction from clot (Ullman et al. 2001). Previous studies indicated that right atrial abnormality has been reported in 2%–30% of PTE patients (Rodger et al. 2000; Weber and Phillips Jr 1966).

Our results illustrated that ST segment depression was a common finding on the right‐sided precordial leads in our patients (26.7%), especially patients with severe RV systolic dysfunction. Furthermore, nearly 87.0% of patients were in normal sinus Rhythm.

### 3.1 Limitations of the Study

Several limitations of this study can be addressed. First, the nature of the study design (cross‐sectional) did not allow for further evaluation of any apparent associations over time. Second, our experience at a single hospital may not be applicable to the broader community. Last, the interpretation of the findings is constrained by the small sample size. Longitudinal studies with larger sample sizes are necessary to investigate the ECG findings of right‐sided precordial leads in patients with RV dysfunction.

## 4 Conclusion

This study revealed that the qR pattern, as well as the rS and QS patterns in V3R and V4R, was significantly more common in patients with severe and nonsevere RV systolic dysfunction, respectively (*p* = 0.0001). The most prevalent ECG feature observed was a deep negative T wave in the right precordial leads (V3R, V4R; 90.0%) in both groups. Therefore, it is recommended that right‐sided precordial leads (V3R, V4R) be utilized in all patients with RV systolic dysfunction. This straightforward approach can aid in the early detection and risk stratification of patients presenting with RV systolic dysfunction.

## Author Contributions

**Reza Khosravi:** conceptualization, review and editing, supervision. **Hasan Shemirani:** methodology, review and editing. **Marziyeh Najafi:** methodology, review and editing. **Zahra Ghaffarinejad:** writing the original draft, review and editing. **Mahta Arbabi:** writing the original draft, review and editing. **Marzieh Tajmirriahi:** conceptualization, review and editing, supervision.

## Consent

Informed consent was obtained from all participants included in this study. Patient confidentiality and privacy were strictly maintained throughout the research process.

Consent for publication: The authors guarantee that the manuscript will not be published elsewhere in any language without the consent of the *Annals of Noninvasive Electrocardiology* journal, that the rights of third parties will not be violated, and that the publisher will not be held legally responsible should there be any compensation claims.

## Conflicts of Interest

The authors declare no conflicts of interest.

## Data Availability

The data that support the findings of this study are available upon request.
